# Dentate gyrus is needed for memory retrieval

**DOI:** 10.1038/s41380-024-02546-0

**Published:** 2024-04-12

**Authors:** Alejandro Carretero-Guillén, Mario Treviño, María Ángeles Gómez-Climent, Godwin K. Dogbevia, Ilaria Bertocchi, Rolf Sprengel, Matthew E. Larkum, Andreas Vlachos, Agnès Gruart, José M. Delgado-García, Mazahir T. Hasan

**Affiliations:** 1 Division of Neuroscience, University Pablo de Olavide, Seville, Spain; 2 Achucarro Basque Center for Neuroscience, Leioa, Spain; 3 Max Planck Institute for Medical Research, Heidelberg, Germany; 4 Instituto de Neurociencias, Universidad de Guadalajara, Guadalajara, 44130 México; 5 Health Canada, Ottawa, ON Canada; 6 Neuroscience Institute Cavalieri-Ottolenghi (NICO), University of Turin, Turin, Italy; 7 NeuroCure, Charité – Universitätsmedizin, Berlin, Germany; 8 University of Freiburg, Freiburg, Germany; 9 Ikerbasque – Basque Foundation for Science, Bilbao, Spain

**Keywords:** Neuroscience, Diseases

## Abstract

The hippocampus is crucial for acquiring and retrieving episodic and contextual memories. In previous studies, the inactivation of dentate gyrus (DG) neurons by chemogenetic- and optogenetic-mediated hyperpolarization led to opposing conclusions about DG’s role in memory retrieval. One study used Designer Receptors Exclusively Activated by Designer Drugs (DREADD)-mediated clozapine N-oxide (CNO)-induced hyperpolarization and reported that the previously formed memory was erased, thus concluding that denate gyrus is needed for memory maintenance. The other study used optogenetic with halorhodopsin induced hyperpolarization and reported and dentate gyrus is needed for memory retrieval. We hypothesized that this apparent discrepancy could be due to the length of hyperpolarization in previous studies; minutes by optogenetics and several hours by DREADD/CNO. Since hyperpolarization interferes with anterograde and retrograde neuronal signaling, it is possible that the memory engram in the dentate gyrus and the entorhinal to hippocampus trisynaptic circuit was erased by long-term, but not with short-term hyperpolarization. We developed and applied an advanced chemogenetic technology to selectively silence synaptic output by blocking neurotransmitter release without hyperpolarizing DG neurons to explore this apparent discrepancy. We performed in vivo electrophysiology during trace eyeblink in a rabbit model of associative learning. Our work shows that the DG output is required for memory retrieval. Based on previous and recent findings, we propose that the actively functional anterograde and retrograde neuronal signaling is necessary to preserve synaptic memory engrams along the entorhinal cortex to the hippocampal trisynaptic circuit.

## Introduction

The hippocampus plays a crucial for encoding, storing, and retrieving memories, such as those found in classical trace eyeblink conditioning, a prototypical model to study declarative memory [1], the association of spatial and sensory cues [2], and conflict resolution [3]. It comprises adjacent cortical regions: the dentate gyrus (DG), CA3, CA2, and CA1 [4–6]. In the DG, new memories are distinguished from the older ones, such as for spatial and contextual representation [7, 8] for pattern separation [9] and encoding, retrieval, and discrimination of episodic memories [10]. In the CA3, recurrent synaptic connections are formed, which appear necessary for pattern completion and memory recall [11]. Environmental cues induce activation of cell assemblies that constantly adapt to changes in external signals and participate in pattern completion, facilitated by EC-CA3 and EC-CA1 synaptic interactions to print memory traces across the EC-trisynaptic circuits [12, 13]. Concurrent EC input to the dendrites of the DG granule cells [14, 15] in concert with pre- and post-synaptic NMDA receptors is required for plasticity [16, 17]. This emerging evidence suggests that memory engrams could be distributed throughout the brain [1, 18, 19].

To investigate whether DG plays a role in memory retrieval, a previous study used Designer Receptors Exclusively Activated by Designer Drugs (DREADD)-mediated clozapine N-oxide (CNO)-induced hyperpolarization [20] to inhibit the spiking activity of DG cells. Remarkably, the study reported that the memory was erased (after hyperpolarizing DG cells for several hours), suggesting that DG is not needed for memory retrieval [21]. It was thus concluded that DG is required for memory maintenance. Notably, another study suggested the opposite with optogenetic-mediated hyperpolarization of DG cells for only a few minutes, namely that the DG is required for memory retrieval [22].

It is conceivable that DREADD-mediated hyperpolarization of DG blocked both anterograde and retrograde neuronal signaling over a long-term period (several hours), which was sufficient to erase the memory engrams along the EC-trisynaptic circuits, without leaving an intact copy to re-establish a memory engram once DREADD was switched-off. On the other hand, light-induced hyperpolarization of the DG by optogenetics did not erase the memory engrams enabling memory retrieval when the light was switched-off. The differences in these two procedures could be due to the duration of hyperpolarization: DREADD/CNO-induced hyperpolarization can last several hours [21, 23]. In contrast, optogenetic-induced hyperpolarization in the study was faster and lasted only a few minutes [22].

Considering the results obtained with these two procedures, we hypothesized that silencing DG synaptic output without hyperpolarizing them would not erase the memory engram even if performed over several days, and memory retrieval would be enabled after un-silencing of synaptic transmission. To test this idea, we developed the next-generation technology based on tetanus toxin light chain (TeTxLC) for virus-delivered Inducible Silencing of Synaptic Transmission (vINSIST-2) [24]. TeTxLC specifically cleaves synaptobrevin-2, a key vesicular protein involved in evoked synaptic transmission for selective silencing of the presynaptic output only. With the vINSIST-2 system, we targeted the DG in alert-behaving rabbits. We used the trace eyeblink conditioning paradigm to investigate whether the memory engram would be either erased as in the previous study with DREADD/CNO-mediated DG inhibition [21] or can be reactivated (retrieved) as in the other study with optogenetic-mediated DG inhibition [22]. Our results show that silencing DG output with vINSIST-2 [24] does not erase the memory engram, which is reactivated for memory retrieval after un-silencing synaptic transmission.

Both previous methods [21, 22] based on neuronal hyperpolarization affect the electrical state of all the targeted DG neurons. This implies that the activity manipulation was generalized, unspecific, and unlocalized, involving many mechanisms beyond synaptic release. The vINSIST method [24] is designed to functionally disconnect circuits by selectively interrupting the neurotransmitter release mechanism, which is a specifically localized and more subtle targeted manipulation directed selectively to interfere with the synaptic output of these cells.

## Results

### Targeting the vINSIST-2 system to the rabbit dentate gyrus for chemically controlled silencing and unsilencing of synaptic transmission

**Fig. 1:**
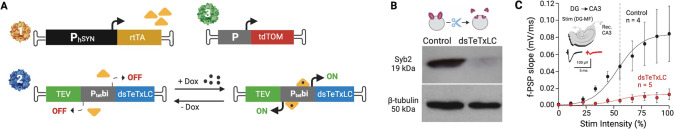
Genetic technology for virus-delivered silencing of synaptic transmission. A Schematic diagram depicting the tetracycline-controlled genetic switches to express a destabilized tetanus toxin light chain (dsTeTxLC) for selective cleavage of a key synaptic vesicle protein, synaptobrevin-2 (Syb2). Virus-1 is equipped with the human synapsin promoter (P_hSYN_) to express the reverse tetracycline transactivator (rtTA), virus-2 has a bidirectional tetracycline promoter (P_tet_bi) to express dsTeTxLC with a short half-life time. Only in the presence of doxycycline (Dox), P_tet_bi is switched-on to express dsTeTxLC. Without Dox, the system is switched-off. For validating targeted gene expression in the brain, the virus-3 was used as a tracer to express tdTomato (tdTOM) under control of P_hSYN_. B Levels of synaptobrevin-2 were determined by a Western blot: without and with dsTeTxLC expression in neurons to validate dsTeTxLC-mediated cleavage of synaptobrevin-2. The beta-tubulin was used for normalization. C Hippocampal slices with stimulating electrode in the dentate gyrus (DG) and recording electrode in CA3 (insert inside the input/output curve). The input/output curves compare the slopes of averaged field potentials (10 repetitions/intensity) with increasing stimulation intensities from control (in black) and TeTxLC-infected (in red) slices (RM-ANOVA test, P < 0.001.

We have developed the next-generation system for with recombinant adeno-associated viruses (rAAVs) for virus-delivered silencing of synaptic transmission or vINSIST with a destabilized TeTxLC (dsTeTxLC; vINSIST-2) consisting in three rAAVs: virus-1 (rAAV-**P**_hSYN_-rtTA), virus-2 (rAAV-**P**_tet_bi-dsTeTxLC/TEV), and virus-3 (rAAV-**P**_hSYN_-tdTOM). The destabilized dsTeTxLC has a half-life of ~5 min (manuscript in preparation). Virus-1 expresses the reverse tetracycline transactivator (rtTA) with a human synapsin promoter fragment (**P**_hSYN_). Virus-2 is equipped with a bidirectional tetracycline (tet) promoter (**P**_tet_bi) to express dsTeTxLC. Virus-3 expresses tdTomatoe (tdTOM) under a **P**_hSYN_ serving as a tracer for documenting precise targeting to the rabbit dentate gyrus. Virus-3 (rAAV-**P**_hSYN_-tdTOM) alone was used as a control. In the presence of Dox, the rtTA/Dox complex binds to the **P**_tet_bi to express dsTeTxLC, whereas without Dox, **P**_tet_bi is switched-off, and dsTeTxLC expression returns back to the baseline level (Fig. 1A). With the rtTA system, gene expression can be fully induced with a single intraperitoneal Dox injection already after 24 h, and expression subsides to baseline levels within 10 days [25, 26]. The dsTeTxLC is a zinc-dependent protease that selectively cleaves the synaptic vesicle protein synaptobrevin-2 (Fig. 1B) as demonstrated by Western blot analysis. Synaptobrevin-2 depleted synaptic vesicles are unable to perform calcium-dependent neurotransmitter release, thus blocking synaptic transmission as shown by synaptic input/output responses by stimulation of the dentate gyrus mossy fibers and recording excitatory field potentials in the CA3 region (Fig. 1C) [24].

### Reversible control of DG synaptic output by dsTeTxLC for memory retrieval

**Fig. 2:**
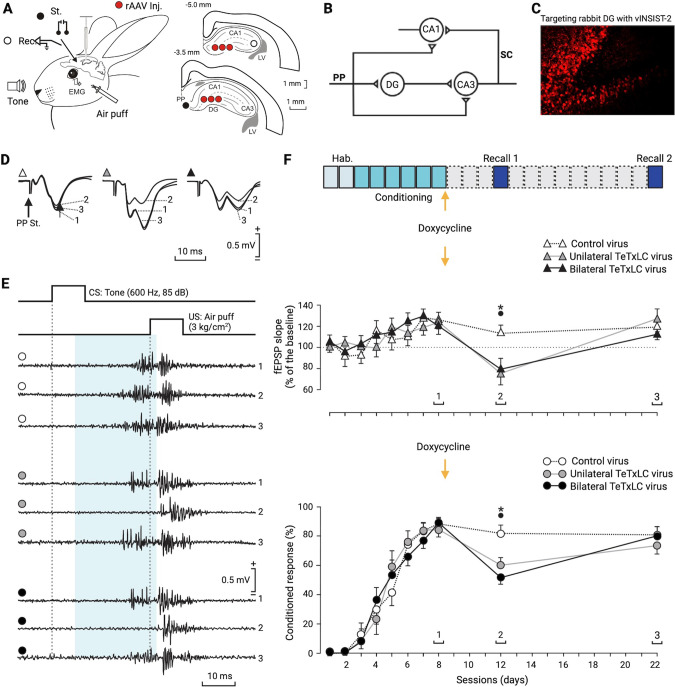
Effects of dentate gyrus synaptic output block and unblock in memory retrieval. A Schematic view of the electrode placements in two regions of the dorsal hippocampus with a stimulation electrode (black filled circles) in the perforant path (PP) to the dentate gyrus (DG) and the recording electrodes in CA3 (open circles). Rabbits were injected in the DG with a cocktail solution consisting of three viruses in 6 different sites as indicated (red filled circles). LV = left ventricle. B Schematic view of the cortical-hippocampal trisynaptic circuit depicting the different pathways, including the PP (EC to DG) and the SC (CA3 to CA1) pathways. C The rAAV cocktail was injected into the rabbit DG and precise targeting was validated by tdTomato (tdTOM) expression (red fluorescence). D Representative examples of fEPSPs (averaged 5 times) evoked at the CA3 area by electrical stimulation of the perforant pathway (PP St.) during the CS-US (interval 200 ms after CS presentation). The traces labeled as 1, 2 and 3 correspond to measurements after conditioning before Dox (without dsTeTxLC expression), during Dox (with dsTeTxLC expression) and after Dox washout (without dsTeTxLC expression). E Evolution of conditioned eyeblink responses evoked in control (white circles), and in unilaterally (gray circles) or bilaterally (black circles) injected animals with Dox. Note the absence of conditioned responses during the period of dsTeTxLC activation (2) as compared with previous (1) and later (3) recording sessions. F From top to bottom is illustrated the experimental design, a quantitative analysis of the evolution of the second component of fEPSPs evoked at the CA3 area by electrical stimulation of the PP, and learning evolution across habituation (days 1-2), conditioning (days 3-8), and recall (days 12 and 22) sessions. Representative examples of recorded fEPSPs and conditioned eyelid responses are illustrated in D and E, respectively. Note that dsTeTxLC activation prevented the proper expression of the expected changes in synaptic strength and learning rates. (*; control vs. unilateral dsTeTxLC inj.), (black dot; control vs. bilateral dsTeTxLC inj.), P ≤ 0.05.

We stereotaxically injected a three-virus cocktail into the rabbit DG (Fig. 2A, C), a crucial region of the hippocamnpus trisynaptic circuit (Fig. 2B). With a tracer virus expressing tdTOMATO (tdTOM), a red fluorescent protein, under the control of the P_hSYN_, we could validate precision targeting (Fig. 1A, 2C). To determine the presence of conditioned eyelid responses, animals were implanted with a bipolar electrode in the upper eyelid to record its electromyographic (EMG) activity (Fig. 2A). To record conditionally evoked changes in synaptic strength, the same animals were also implanted with a bipolar stimulating electrode in the perforant pathway and with a tetrode for recording in the ipsilateral CA3 area (Fig. 2A). Classical trace eyeblink conditioning paradigm was performed on rabbits with virus targeted to the DG (Fig. 2A, C) [1, 21, 27, 28].

In awake behaving rabbits, the electrical stimulation of the perforant pathway evoked a field excitatory postsynaptic potential (fEPSP), presenting two successive components corresponding to the monosynaptic activation of the CA3 pyramidal cells and their polysynaptic activation across DG granule cells (Fig. 2A–D). The acquisition of these conditioned responses was accompanied by a small increase in the slope of the evoked fEPSPs at the DG-CA3 synapse (Fig. 2A, D). After two habituation sessions during which the CS was presented alone, animals received six sessions with paired conditioned and unconditioned stimuli (CS-US) presentations (Fig. 2E). Rabbits were conditioned for a maximum of 6 days (Fig. 2F) until they reached the selected criterion for associative learning (>70% of conditioned responses/session). The criterium was reached by the 4th conditioning session (Fig. 2F).

After conditioning sessions, animals from the first group were intraperitoneally injected with Dox. Four days later, animals were re-recorded for a recall session (Fig. 2F). In this situation, rabbits that received either uni- or bilateral tdTOM/TeTxLC injections (*n* = 3 each) presented a significant (Two-way ANOVA, *P* ≤ 0.05) decrease in both the percentage of evoked conditioned responses and on the slopes of the second, long-latency component of the fEPSP evoked at the hippocampal CA3 area by the electrical stimulation of the perforant pathway. In contrast, control animals (*n* = 3) presented values similar (*P* ≥ 0.092) to those reached during the 6th conditioning session (Fig. 2D, E, F).

Since dsTeTxLC-mediated silencing of the DG synaptic output lasts for up to 10 days, we conducted a second recall session 14 days after the Dox injection. Interestingly, by that time, the percentage of conditioned responses and the slope of fEPSPs evoked at the PP-CA3 synapse had reached values similar (*P* ≥ 0.136) to those presented by the 6th conditioning session, i.e., the day before the Dox injection (Fig. 2D, E, F). These results indicated that the transient disconnection and reconnection of DG granule cell mossy axons on hippocampal CA3 only affected the acquired memory during the disconnection period.

### Persistent conditioning does not compensate for memory retrieval during transient DG synaptic output block

**Fig. 3:**
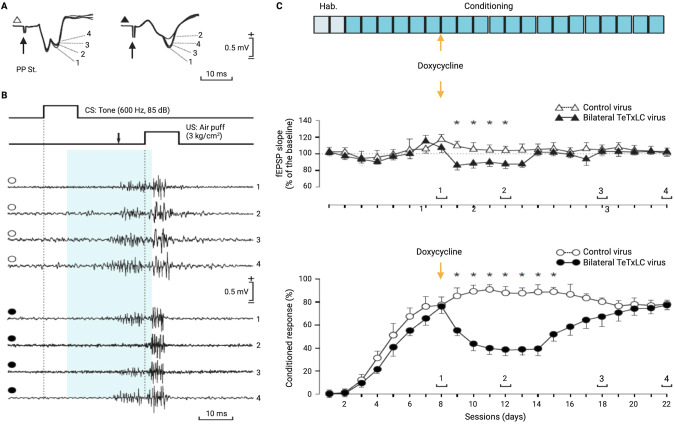
Continuous classical conditioning during a brief period of dentate gyrus synaptic output block and unblock in memory retreival. **A** Representative example of fEPSPs (averaged 5 times) evoked at the PP-CA3 synapse witht the CS-US interval of 200 ms after CS presentation during conditioning. The traces labeled as 1, 2 and 3/4 correspond to measurements before Dox (without dsTeTxLC expression), during Dox (with dsTeTxLC expression) and after Dox washout (without dsTeTxLC expression). **B** Evolution of conditioned eyeblink responses evoked in control (white circles) and in bilaterally (black circles) injected animals with Dox. Note the absence and/or decrease in the amplitude of conditioned responses during the period of dsTeTxLC activation (2) as compared with previous (1) and later (3/4) recording sessions. **C** Illustrated from top to bottom is the experimental design, a quantitative analysis of the evolution of the second component of fEPSPs evoked at the CA3 area by the electrical stimulation of the PP, and learning evolution across habituation (days 1–2), conditioning (days 3-22) sessions. Representative examples of recorded fEPSPs and conditioned eyelid responses are illustrated in (**A**, **B**), respectively. Note that dsTeTxLC activation prevented the proper expression of the expected changes in synaptic strength and learning rates. *P ≤ 0.05.

In a second set of experiments (Fig. 3), we checked whether the transient disconnection between DG granule cells and CA3 pyramidal cells would affect the normal performance of an already acquired conditioned eyelid response and its effects on the concomitant fEPSPs evoked at the CA3 area by perforant pathway stimulation (Fig. 3A, B). Animals with virus targeting the DG (dsTeTxLC) in this group were conditioned for 20 days (Fig. 3C). As shown in Fig. 3C, dsTeTxLC activation by Dox injection in well-trained animals produced a significant (Two-way ANOVA, *P* ≤ 0.05) decrease in the percentage of evoked responses (from the 7th to the 13th conditioning sessions) in those animals (*n* = 3). This decrease in the percentage of conditioned responses was accompanied by a significant (Two-way ANOVA, *P* ≤ 0.05) reduction in the slope of the late component of fEPSPs evoked at the PP-CA3 synapse. Interestingly, those deficits were not compensated during successive conditioning sessions: an improvement started with the 15th session (i.e., the 7th session after the Dox injection) and became fully complete, reaching control values, by the 22nd training session (i.e., 14 sessions after the injection). These results suggest that the performance of an already acquired motor ability during persistent conditioning could also be affected by the experimental disconnection of the DG-CA3 synapse, but that this depressing effect was recovered to control values after this synapse was reconnected. These results also suggest that neither compensatory nor alternative neuroanatomical pathways participate in restoring memory engram by persistent conditioning during the silencing period.

### The density of vGLUT1 and PSD95 expressing puncta decreases after mossy fiber output block with dsTeTxLC

**Fig. 4:**
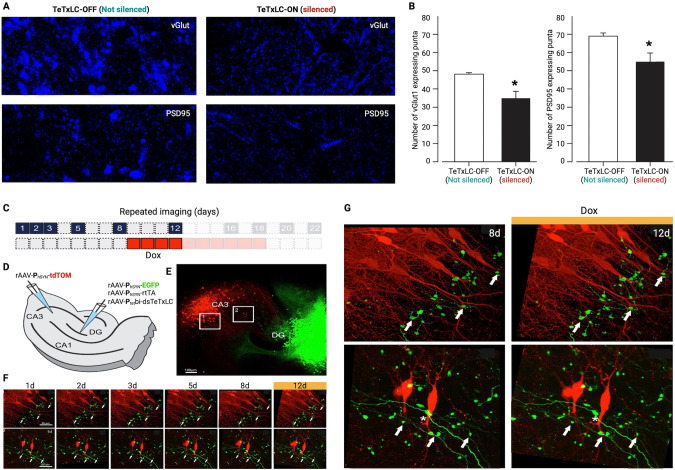
Expression of vGLUT1 and PSD95 puncta and stability of mossy fiber boutons in response to dentate gyrus synaptic output block. **A** Confocal imaging of vGLUT1 and PSD95 expressing puncta in DG mossy fibers and CA3 neuropil of control and dsTeTxLC rabbit brain slices. Note the dsTeTxLC labeled puncta in all four cases. Scale bar: 25 µm. **B** Graphs representing the changes in the density of vGLUT1 (Student’s *t*-test 2.337; *P* = 0.0416) and PSD95 (Student’s *t*-test = 2.345; *P* = 0.0410) expressing puncta in the neuropil of CA3, control group *n* = 5 and dsTeTxLC infected group *n* = 7. Asterisks in bars indicate statistically significant differences from the control group after the student’s *t*-test; *P* ≤ 0.05 (*). Abbreviations: vGLUT1: the vesicular glutamate transporter 1 and PSD95: the postsynaptic density 95. **C** Timeline of repetitive confocal imaging. **D, C** rAAV-assisted precise targeting of DG and CA3 with tdTOM and EGFP in mouse entorhino-hippocampal slice cultures, respectively, Scale bar = 100 µm. **E**, **F** Baseline imaging of DG mossy fiber boutons in the CA3 region of interest (ROI) 1 and 2 (from (**E**) from day-0 (0d) until day-9 (9d) and Dox-induced dsTeTxLC for 6 days (starting after the imaging at day 3 until day 9) is shown (*n* = 5; 125 mossy fiber boutons total; in one experiment 2 out of 25 boutons were lost after Dox-induced dsTeTxLC). Scale bars = 50 µm. **G** High magnification of images of slices without Dox (without dsTeTxLC expression) and with Dox ((with dsTeTxLC expression).

Dox-dependent inducible silencing of synaptic transmission was performed in the hippocampus’s rostral and caudal DG with dsTeTxLC (Fig. 4A, B). The dsTeTxLC-assisted silencing of the granule cells’ mossy *fiber* projections was labeled along the pathway to the CA3 region (Fig. 4A, B). The density of puncta expressing vGLUT1, a presynaptic marker of excitatory buttons [29], and PSD95, an excitatory synapse postsynaptic marker [30, 31], both crucial for the organization of synaptic strengths, were analyzed and compared in layer CA3 of the hippocampus. Animals injected with the dsTeTxLC virus showed a statistically significant decrease in both densities of vGLUT1 expressing puncta (Student’s *t*-test = 2.337; *P* = 0.0416) and PSD95 expressing puncta (Student’s *t*-test = 2.345; *P* = 0.041; Fig. 4A, B). Compared to controls, after the injection of the dsTeTxLC virus to the DG, there was a decrease of ~30% in vGLUT1 expressing puncta, and of ~20% in PSD95 expressing puncta, belonging to the CA3 pyramidal cells.

### DG mossy fibers do not retract after blocking synaptic output

Mouse entorhino-hippocampal slice cultures were prepared, and two sets of viruses were locally injected: DG was targeted with rAAV-**P**_hSYN_-EGFP, rAAV-**P**_hSYN_-rtTA and rAAV-**P**_tet_bi-dsTeTxLC and CA3 with rAAV-**P**_hSYN_-tdTOM (Fig. 4D, E). After 2 weeks, time-lapse confocal imaging sessions were performed in CA3 to visualize DG-MF boutons (Fig. 4C, F, on days indicated in blue). Repetitive baseline imaging showed high stability of EGFP-labeled boutons. After baseline imaging, slices were treated with Dox (1 μg/ml) in the medium to activate dsTeTxLC expression. Even after 6 days under Dox for dsTeTxLC expression, boutons were stably detectable as during the baseline imaging sessions (*n* = 5; 125 mossy fiber boutons total; in one experiment 2 out of 25 boutons were lost after Dox-induced dsTeTxLC expression) (Fig. F, G and data not shown). These results indicate that blocking DG-MF output does not induce the loss of boutons and most likely the DG-MFs to CA3 connections remained intact.

## Discussion

Exploring the roles of interacting brain circuits has been a significant challenge for modern neuroscience. It is becoming increasingly clear that memory engrams are generated in different brain regions [1, 18, 19, 32]. Each brain area has multiple cell types and connectivity patterns within and between brain regions, equipped with molecular signatures, that are constantly shaped by experience [33–35]. Notably, active, and efficient anterograde and retrograde neuronal signaling between neurons establishes and maintains synaptic connections [5, 34, 36–40]. This allows memory engrams to be formed across synaptically-connected brain regions, involving different bidirectional mechanisms, such as the release of various factors, endosomal tracking, cytoskeleton dynamics in neuronal compartments [41], calcium influx [42], neurotrophin factors(s) [43], nitrogen oxide/carbon monoxide [44], 2-arachidonoylglycerol for cannabinoid signaling [45], dopamine transporter trafficking [46], and epigenetic [47] and gene expression [48, 49]. Inhibition of neuronal activity by hyperpolarization can interfere with numerous biochemical processes [42] that, in turn, could disrupt intrinsic [50], synaptic [38], and homeostatic plasticity [51], and thus learning and memory processes.

In the hippocampus, DG serves as a gateway for the flow of information from EC to the hippocampus. The DG granule cells receive input from the EC and provide information along the mossy fiber pathway to the CA3, which projects to the CA1. There are also direct connections from the EC to CA1 (and to CA3). DG-CA3 circuits are essential for pattern separation [9, 10], while CA3-CA1 neurons contribute to pattern completion [11]. Memory engrams are likely formed and maintained along these EC-trisynaptic-subcortical-cortical circuits. It has been suggested that EC inputs to the DG are important for learning but not for recall. On the other hand, direct perforant path EC input to CA3 provides cue-assisted memory recall but is not used for learning [12]. Sensory signals during learning activate cell assemblies in the EC that project to the dendrites of DG granule cells (GCs) [14, 15]. It is plausible that concurrent EC input to the DC-GCs dendrites in concert with presynaptic NMDA receptors on mossy fibers (MF) [52] and postsynaptic NMDA receptors in CA3 neurons [17] induce plasticity [53]. CA1 dendritic activity patterns play a crucial role in place field determination [54]. The complexity of the hippocampus circuits in mapping external and internal synaptic input against the backdrop of intrinsic circuit-driven synaptic activity [6] is further shaped by the interaction between DG-GC axons projecting to the hilus, where they make synaptic connections with mossy cells (MCs). Different classes of GABAergic DG and CA3 neuron types, such as parvalbumin-positive and somatostatin-positive neurons, bidirectionally control synaptic drive to their project to CA3 and between CA3-CA1 and EC-CA1 circuits [55], that modulate synaptic plasticity by excitation-inhibition balance [56]. These processes play important roles in generating stable engrams [57, 58] and temporally binding them across brain regions for memory recall [59–61]. In addition, gap junctions [62] formed in these hippocampal circuits might serve as cellular and network mechanisms for ultrafast communication between cells for assembling memories, their storage, and recall [12].

There are two widely used technologies for inhibiting neuronal activity: DREADD [20] activated by (CNO) and microbial opsins proteins such as halorhodopsin activated by photostimulation. Both methods inhibit target neurons by hyperpolarizing them. Previous studies showed that after learning, optogenetic inhibition of the DG impaired the expression of initially encoded memory, demonstrating necessity [22], whereas optogenetic activation of learning-tagged DG neurons induced memory recall, indicating sufficiency [63]. Whether memory trace remained stable in the hippocampus circuitry or moved to the cortex over time is at the beginning stage of our understanding [64, 65]. Interestingly, it was reported that virus-delivered DREADD/CNO-mediated inhibition of the DG after trace eyeblink conditioning erased the memory engrams along the EC-trisynaptic circuits [20]. It is, however, possible that memory engrams were suppressed or had become latent by prolonged hyperpolarization [66, 67]. Notably, the optogenetic-induced hyperpolarization of DG neurons was applied for a few minutes [22], while DREADD-induced hyperpolarizing lasted for at least a few hours [21, 23].

We suggest that DREADD/CNO-induced hyperpolarization was sufficient to erase the synaptic memory engrams from the DG-CA3 synapse in both the anterograde direction (presynaptic DG to postsynaptic CA3) and retrograde direction (postsynaptic DG to presynaptic EC), thus erasing memory engrams along the EC – hippocampus trisynaptic circuits. Transient hyperpolarization by optogenetic (by light illumination) was instead short enough to retain the engram, which led to memory retrieval when light illumination was switched-off. We suggest that these two opposite findings were likely due to the different technologies applied.

To clarify the issue, we hypothesized that if DG synaptic output is silenced selectively, without hyperpolarizing neurons, by blocking presynaptic neurotransmitter release, the synaptic memory engram would not be erased. In this way, memory retrieval would be re-established after the un-silencing of synaptic transmission. To selectively block DG output, we developed and applied the next-generation advanced chemically controlled technology for virus-delivered Inducible Silencing of Synaptic Transmission version-2 (vINSIST-2) (Fig. 1). Previous studies have shown that TeTxLC-mediated DG-MF silencing of synaptic output leads to normal MF excitability, normal MF projection throughout CA3 stratum lucidum, unaltered ultrastructural MF terminals, unaltered electrophysiological characteristics, and normal LTP of perforant path inputs [68]. This makes our TeTxLC-based vINSIST-2 system highly valuable to investigate the selective role of DG output without hyperpolarizing neurons.

To explore this possibility, we used the trace eyeblink conditioning paradigm, which is considered a prototypic example of declarative memory, and we used rabbits for our experiments because it is a well-established animal model for trace eyeblink conditioning [69] with air-puff as an US (without the need to perform electrical stimulation to the eyelid as commonly done with mice, which can activate additional somatosensory circuits). The rabbits were trained in trace eyeblink conditioning consisting of a tone followed by an air puff to the eye’s cornea with a 500-ms temporal interval. This training resulted in robust memory formation for the conditioned eyeblink response to the tone when presented alone. We used the vINSIST-2 to target the rabbit DG to selectively block synaptic transmission of the DG-MF output. Three weeks after vINSIST-2 delivery to the DG, we performed conditioning. In vivo electrophysiology recordings were carried out at the DG-CA3 synapse to measure network-level development of synaptic strength over the conditioning trials while also measuring conditioned responses (Figs. 2, 3). Our results showed that synaptic connections between the DG-MF and CA3 were strengthened during conditioning following increased conditioned responses. Silencing of synaptic transmission of the DG-MF over days by Dox-induced vINSIST-2 expression reduced conditioned responses, and the synaptic strengths between DG-MF and CA3 neurons collapsed. However, un-silencing DG-MF synaptic transmission by removing Dox for 2 weeks, mainly due to slow Dox clearance from the brain [25, 26], restored conditioned responses and the synaptic strengths between DG-MF and CA3 (Fig. 2). Similar results were found during over-conditioning, where the conditioning sessions continued after Dox-induced vINSIST-2 expression during the silencing session, and learning level and synaptic activity were recovered entirely 2 weeks after the Dox injection (Fig. 3), demonstrating that the previously formed memory trace or engram was not erased. Our results show that despite of animals being “overtrained”, no increase in the number of conditioned responses was observed during prolonged silencing of the synaptic transmission, and memory retrieval was enabled after unsilencing of synaptic transmission. The DG-MF output was therefore required for memory retrieval, and furthermore, no new engrams could overtake with persistent conditioning during the silencing period.

The dynamics organization of synaptic proteins are thought to be crucial for sensory processing and learning and cognition [70–75]. In support of our hypothesis, we found that upon silencing of synaptic transmission, vGLUT and PSD95 puncta corresponding to the DG-MF boutons and CA3 spines were significantly reduced (Fig. 4A, B). In keeping with this hypothesis is the finding that activity-dependent recruitment of synaptic proteins for efficient synaptic transmission plays a pivotal role in preserving and stabilizing the synaptic memory engram(s) [73, 76]. Reduction of vGLUT and PSD95 at the DG-CA3 synapse, as detected in our study by silencing of synaptic transmission, functionally disconnected the synaptic engram, which upon un-silencing the engram was recovered, possibly by activity-dependent recruited vGLUT and PSD95 back to the synaptic connections.

To investigate whether vINSIST-2 assisted silencing of DG output induces the loss of MF boutons in mouse entorhinal-hippocampal slice cultures, we used vINSIST-2 to the DG and virus-delivered green fluorescence protein (EGFP) and tdTOM to DG and CA3, respectively. We performed time-lapse repetitive confocal imaging on slice cultures over several days to establish stability of DG-MF boutons projecting to the CA3 region of the hippocampus. We found that even after 6 days of Dox-induced dsTeTxLC-mediated silencing of synaptic transmission DG-MF boutons remained stable (Fig. 4C–G**)**, consistent with previous findings [68, 77].

**Fig. 5:**
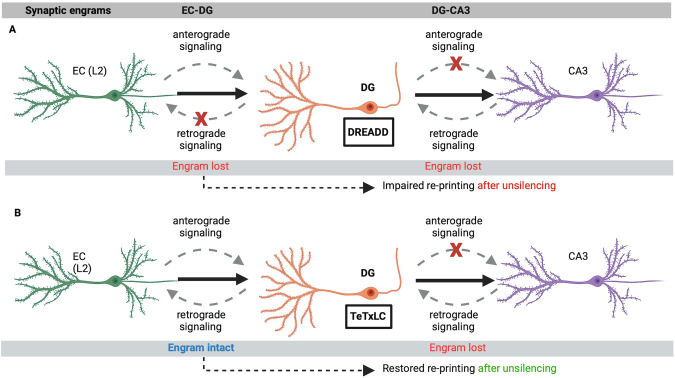
Schematic diagram depicting a hypothesis for the sequential printing of memory engrams along the EC-trisynaptic circuit. **A** DREADD-mediated DG neuronal hyperpolarization due to anterograde and retrograde inhibition erases both EC-DG and DG-CA3 synaptic engrams, without leaving an intact synaptic engram. **B** With dsTeTxLC-mediated silencing of DG mossy fiber output, the EC-DG synaptic engram remains intact, while the DG-CA3 synaptic engram is erased. After un-silencing of synaptic transmission, the the intact EC-DG synaptic engram is re-printed to establish the DG-CA3 synaptic engram, thus enabling memory retrieval.

We suggest that, unlike the DREADD-mediated inhibition of DG, that impaired memory expression, possibly by erasing the EC-DG and DG-CA3 synaptic engrams along the EC-trisysnaptic circuit, the silencing of DG-MF output by our vINSIST-2 (dsTeTxLC) technology, would keep the synaptic engram undisturbed and intact for memory retrieval after un-silencing of synaptic transmission (Fig. 5). The differences in the results are likely due to the genetic approaches used; the DREADD system hyperpolarizes neurons, blocking anterograde and retrograde signaling, while dsTeTxLC only blocked presynaptic neurotransmitter release by cleavage of vesicle-containing synaptobrevin-2 for evoked synaptic transmission. Thus, in the previous work with DREADD-mediated DG inhibition [21], the memory trace that develops in the DG possibly functionally disconnected between the presynaptic neurons providing input to the DG and the postsynaptic neurons that receive information from the DG. With such polysynaptic functional disconnection, we suggest that synaptic memory engrams previously formed along the EC-DG-CA3 pathway were erased. However, with vINSIST-2 (dsTeTxLC)-mediated presynaptic silencing of the DG-MF output, the memory engram remained undisturbed and intact for retrieval after un-silencing of synaptic transmission.

It is important to note that synaptic networks are equipped with mechanisms that can recruit a repertoire of synaptic connections and weights for flexibility and efficient formation of sensory representation and memory retrieval [78–81]. Considering previous findings [21, 22], our current work suggests that memory engrams are synaptically printed from one region to the next and distributed throughout the brain. It seems quite plausible that destabilization of synaptic connections after silencing and subsequent un-silencing of synaptic transmission would flexibly establish re-organized synaptic connectivity and synaptic weights [78–80]. Based on dynamically activated cell assemblies [81] in EC, DG, CA3, CA1, and cortical regions, and synaptically connected subcortical and cortical areas, and previous findings [18, 19], and supported by our results, it seems likely that memory engrams are sequential printed from one synaptic connection to the next across the different brain regions [32].

All methods are helpful, but our interpretation of experiments must carefully consider the mechanisms and differences across these procedures to better understand how biology and the brain work. The discoveries made with the application and DREADD and optogenetics and our vINSIST-2 technologies to investigate the role of DG in memory retrieval have provided significant insight based on the circuit manipulation schemes [21, 22, 24]. A comprehensive understanding of the EC-trisynaptic and subcortical and cortical circuits would continue to demand the development of advanced genetic tools for slice electrophysiology and in vivo imaging, recordings, and activity manipulation of different cell types to dissect their role and gain deeper insight into the learning and memory processes.

## Materials and methods

### Animals

Experiments were carried out on adult male rabbits (New Zealand white albino) weighing 2.6–3.1 kg on arrival, obtained from an authorized supplier (Isoquimen, S.L., Barcelona, Spain). Rabbits were housed in individual cages for the experiment and kept on a 12/12 h light/dark cycle with constant ambient temperature (21 ± 1 °C) and humidity (50 ± 7%). Food and water were available *ad libitum*. All experimental procedures followed the guidelines of the European Union Council (2010/276:33-79/EU) and Spanish (BOE 34:11370-421, 2013) regulations for using laboratory animals in chronic experiments. The local University Ethics Committee also approved experimental protocols.

The study involved a total of 10 male C57BL/6 wild-type mice obtained from Charles River (Sulzfeld, Germany). The mice were group-housed in cages (type III, 825 cm^2^; Ehret, Emmendingen, Germany) with a maximum of 5 mice per cage, and they had *ad libitum* access to food and water. The experiments were conducted at the Max Planck Institute for Medical Research, adhering to the animal welfare guidelines of the Max Planck Society and the studies received approved by the regional commission in Karlsruhe (G-171/10).

### AAV plasmids

We developed the next-generation genetic technology for virus-delivered inducible silencing of synaptic transmission version-2 (INSIST-2). Two plasmid sets were generated, and recombinant adeno-associated viruses (rAAVs) were prepared with the tetracycline-controlled genetic switches: Set-1 included rAAV-**P**_hSYN_-tdTOMvirus, meanwhile, Set-2 included the rAAV-**P**_hSYN_-rtTA, rAAV-**P**_tet_bi-dsTeTxLC/TEV and rAAV-**P**_hSYN_-tdTOM viruses. The destabilized tetanus toxin (dsTeTxLC) has a very short half-life time. The vINSIST-2 system will be described in a separate publication.

### Protein extraction and Western blot

Rat organotypic slices were prepared as described previously [82]. Slices were infected with either rAAV-**P**_tet_bi-dsTeTxLC/TEV and rAAV-**P**_hSYN_-tTA were harvested in cold lysis buffer (50 mM Tris-HCl, pH 7.6; 5 mM MgCl_2_; 130 mM NaCl; 10 mM KCl; 1% Triton X-100; 5% Glycerin) 14 days after virus infection and homogenized by sonication. Lysis buffer was supplemented with a protease inhibitor cocktail (Complete^TM^; Roche). Protein concentration was determined by Bradford assay. 15 µg of the protein lysates from both rAAV infected and uninfected rat hippocampus organotypic slices were separated by SDS-Page gel (15% separating and 6% stacking gels) and transferred onto nitrocellulose membranes. Western blots were probed with the following primary antibodies: synaptobrevin-2 (1:1000, Abcam) and polyclonal mouse anti- ß-tubulin (1:1000, Millipore). The secondary antibodies used were; horseradish peroxidase-linked anti-rabbit, anti-goat, or anti-mouse (1:15000, Vector Laboratories, Peterborough, UK). Western blots were detected by an enhanced chemiluminescence kit (ECL kit, Amersham Pharmacia Biotech, Freiburg, Germany).

### Electrophysiological Experiments

Entorhinal cortex–hippocampus transverse slices (400 µm) were obtained from 6-week-old mice, as described before [83]. The dissection buffer consisted of 212.7 mM sucrose, 5 mM KCl, 1.25 mM NaH_2_PO_4_, 10 mM MgCl_2_, 0.5 mM CaCl_2_, 26 mM NaHCO_3_, and 10 mM dextrose. Following storage in regular artificial cerebrospinal fluid (aCSF) at room temperature, the slices were transferred to a recording chamber and perfused with aCSF at 30°C ± 1 °C. The aCSF shared similarities with the dissection buffer, with the substitution of sucrose by 119 mM NaCl, a reduction of MgCl_2_ to 1 mM, and an increase in CaCl_2_ to 2 mM. Both the dissection buffer and aCSF were saturated with 95% O_2_/5% CO_2_ (pH 7.4). Extracellular responses from area CA3b to DG stimulation were recorded, filtered, and digitized using an EPC-10 amplifier. Input/output curves were obtained, and statistical analysis was performed using MATLAB. Results are reported as averages ±SEM, with significance set at *P* ≤ 0.05.

### Animal handling and stereotactic vINSIST-2 virus injection in the rabbit brain

To accomplish full DG targeting, we injected 9 points of the dentate gyrus for unilateral infected animals, and 18 points for bilateral infected animals using the corresponding set of viruses. For this purpose, the needed number of points were drilled in the skull of the animal during the surgery for electrode implantation, and the cocktail of the virus was delivered using a glass pipette connected to a plastic tube and, finally, to a 50 ml syringe that was used as an impulsion system. A total volume of 0.75 µl was injected at each of the selected sites at a speed of 0.15 µl/min. The stereotaxic coordinates used to spread the infection over the whole dentate gyrus are shown in the supplementary information (Table 1).

Here we applied an advanced next-generation technology for **v**irus-delivered **In**ducible **Si**lencing of **S**ynaptic **T**ransmission or vINSIST-2. The dorsal DG of rabbits was injected with a solution consisting of three viruses (0.75 µL in 9/18 different points). A single intraperitoneal Dox injection in rabbits allows for neuron-specific dsTeTxLC expression, which cleavages synaptobrevin-2, impairing synaptic transmission. As brain Dox concentration subsides, dsTeTxLC expression is also reduced in parallel, restoring normal synaptic transmission. Experimental groups: in the first series of experiments (*n* = 3 animals per group; Fig. 2), animals received two habituation (days 1–2) and six conditioning (days 3–8) sessions. Afterward, they received two recall sessions (days 12 and 22). In the second series of experiments (*n* = 3 animals per group; Fig. 3), animals received two habituation and 20 conditioning sessions. In both cases, animals received Dox injection 30 min before the 9th session. As brain Dox concentration subsides, dsTeTxLC expression is also reduced in parallel, restoring normal synaptic transmission.

In all the animals, the dsTeTxLC expression was activated by doxycycline (Dox) injection after 6 days of conditioning (Figs. 2 and 3). In the first series of experiments (Fig. 2), two recall sessions were carried out 4 and 14 days after doxycycline injections. In the second series of experiments (Fig. 3), animals were conditioned for 14 additional days after doxycycline injections.

At the end of the experiments, animals were deeply anesthetized with sodium pentobarbital (50 mg/kg, i.p.) and perfused transcardially with saline and 4% paraformaldehyde. To determine the final location of recording and stimulation sites, the brains were removed from animals and cut into slices (50 µm). The relevant brain areas were processed for Nissl (toluidine blue) staining.

### Classical conditioning of eyelid responses and fEPSP recordings in behaving rabbits

Experiments were carried out on adult male rabbits (New Zealand white albino) weighing 2.6–3.1 kg on arrival, obtained from an authorized supplier (Isoquimen, S.L., Barcelona, Spain). Animals were housed in individual cages for the experiment and kept on a 12/12 h light/dark cycle with constant ambient temperature (21 ± 1 °C) and humidity (50 ± 7%). Food and water were available *ad libitum*. All experimental procedures followed the guidelines of the European Union Council (2010/276:33-79/EU) and Spanish (BOE 34:11370-421, 2013) regulations for using laboratory animals in chronic experiments. The local University Ethics Committee also approved experimental protocols.

Classical conditioning was carried out using a trace paradigm. Animals were presented with a tone as a conditioned stimulus and an air puff as an unconditioned stimulus. Conditioned responses were determined from the EMG activity of the orbicularis oculi muscle. Animals were prepared for the chronic recording of fEPSPs evoked at the CA3 area of the dorsal hippocampus by the electrical stimulation of the ipsilateral perforant pathway and for the classical conditioning of eyelid responses. For this, they were anesthetized with a ketamine-xylazine cocktail (Ketaminol, 50 mg/mL; Rompun, 20 mg/mL; and atropine sulfate, 0.5 mg/kg) and implanted with bipolar stimulating electrodes in the perforant pathway and with single tetrode recording electrodes (homemade tungsten electrode with 4 tips separated by 0.2 mm) in the hippocampal CA3 area. Stimulating and recording electrodes were made from 50 µm, Teflon-coated tungsten wire (Advent Research Materials Ltd., Eynsham, England). The impedance of recording electrodes was always ≤1 MΩ. The final position of hippocampal stimulating and recording electrodes was determined under recording procedures until a reliable monosynaptic field EPSP was identified [6, 27, 28]. During the same surgical step, animals have injected uni- or bilaterally (*n* = 3 per group) in the dorsal DG with the viral Set-2. Additional control animals (*n* = 3 per group) were injected with the viral Set-1. All the animals were also implanted with recording bipolar hook electrodes in the left orbicularis oculi muscle to record its EMG activity. These electrodes were made from Teflon-coated stainless-steel wire (A-M Systems, WA, USA) with an external diameter of 50 μm. A silver electrode (1 mm in diameter) was attached to the skull (occipital bone) as a ground. All wire connections were covered with cyanoacrylate glue, and the whole system was attached to the skull with three small screws fastened and cemented with an acrylic resin to the bone [6, 28]. Terminals of hippocampal stimulating and recording, EMG, and ground electrodes were soldered to nine-pin sockets.

Conditioning consisted of two habituation and 6 conditioning sessions in the case of the recall protocol and 20 conditioning sessions in the case of the over-conditioning protocol. The trace conditioning paradigm consisted of a 100 ms, 600 Hz, 85 dB tone followed 300 ms after CS onset by a 100 ms, 3 kg/cm2 air puff aimed at the left cornea; thus, a trace interval of 200 ms was left between CS end and US onset. Conditioning sessions consisted of 66 trials (6 series of 11 trials each) separated randomly by intervals of 50–70 s. Of the 66 test trials, six were presented in the CS alone. A complete conditioning session lasted for ~1 h. The CS was presented alone during habituation sessions for the same number of blocks/sessions and trials/blocks. As a criterion, we considered a “CR” the presence, during the CS-US interval, of EMG activity lasting >10 ms and initiated >50 ms after CS onset. In addition, the integrated EMG activity recorded during the CS-US interval had to be at least 1.2 times greater than the integrated EMG recorded immediately before the CS presentation. As a criterion for learning, animals should evoke >70% CRs by the 10th conditioning session [6, 27, 28, 84].

### Recording and stimulation

Two weeks after surgery, rabbits were placed in a Perspex box designed to limit the subject’s movements [6, 27, 28, 84]. The box was placed on the recording table and covered by a black cloth. The recording room was softly illuminated, and a 60-dB background white noise was switched-on during the experiments. The EMG activity of the selected muscle was recorded using Grass P511 differential amplifiers with a bandwidth of 0.1 Hz to 10 kHz (Grass-Telefactor, West Warwick, RI, USA). The fEPSPs were recorded with a 16-channel extracellular differential AC amplifier (Model 3500, A-M Systems, Sequim, WA, USA) provided with a head-stage interface adapter. Air puffs aimed at the left cornea were applied through the opening of a plastic pipette (3 mm in diameter) attached to a metal holder fixed to the animal’s nine-pin socket (Dual-channel air-puff device, Biomedical Engineering Co.). Tones were applied from a loudspeaker 80 cm below the animal’s head. Electrical stimulation of the selected sites was achieved with a CS-220 stimulator across an ISU-220 isolation unit (Cibertec, Madrid, Spain). Single (cathodal, square, 50 ms, <1 mA pulses) or paired (40 of an inter-pulse interval) stimuli were programmed.

### Fluorescence Immunohistochemistry

Five rabbits were used for the control group, and 7 rabbits were used to analyze the puncta density after injected dsTeTxLC expression. Brains for fluorescence immunohistochemistry were cryoprotected with 30% sucrose in PB. Then coronal sections (50 μm) were obtained with a sliding freezing microtome (Leica SM2000R) and stored at –20 °C in 30% glycerol and 30% ethylene glycol in PB until used. Brain slices were processed “free-floating” for immunohistochemistry, and all the sections studied passed through all procedures simultaneously to minimize any difference from immunohistochemical staining itself. To analyze the density of the vesicular glutamate transporter 1 (vGLUT1) expressing puncta, a presynaptic marker of excitatory boutons [29], and the postsynaptic density 95 (PSD95) representing puncta, an excitatory synapse postsynaptic markers [71, 85], we have performed immunohistochemistry using primary antibodies against VGLUT1 or PSD95 (Millipore Iberica S.A.U, Madrid, Spain). Briefly, sections were incubated for 1 h with 5% normal donkey serum (NDS) (AbDSerotec, MorphoSys, Kidlington, UK) in phosphate-buffered saline (PBS) with 0.2% Triton-X-100 (Sigma-Aldrich, St. Louis, MO, USA) and then they were incubated overnight at room temperature with mouse monoclonal IgG anti-vGLUT1 (1:1000) or mouse monoclonal IgG anti-PSD95 (1:700) with PBS containing 0.2% Triton-X-100 and 3% NDS. On the second day, sections were washed and incubated for 1 h with anti-mouse IgG secondary antibodies generated in donkeys and conjugated with Alexa 488 and Alexa 647 (1:200; Millipore Iberica S.A.U, Madrid, Spain) in PBS containing 0.2% Triton-X-100 and 3% NDS. Finally, sections were mounted on slides and cover-slipped using Prolong Gold antifade reagent fluorescent mounting medium (Millipore Iberica S.A.U, Madrid, Spain).

### Analysis of vGLUT1 and PSD95 expressing puncta

All slides were coded before analysis, and the codes were not broken until the experiment was finished. The density of vGLUT1 and PSD95 expressing puncta were analyzed and compared in the CA3 region of the hippocampus. Sections from the same rostral-caudal level were examined under a confocal microscope (Leica TCS SPE). Z-series of optical sections (0.5 μm apart) were obtained using sequential scanning mode and processed with ImageJ software. Photographs were taken at 63× magnification. The values of acquisition settings, such as the laser intensity percentage, gain, offset, and resolution, were identical for each stack taken from the same level. All of them had a similar time of exposure to the confocal laser. Subsequently, confocal images from similar Z-position in which the same level of antibody penetrability was observed were chosen from each stack, and five random sampling with size 16 × 16 μm were collected to analyze to avoid somas or blood vessels. Then, the background fluorescence of each image was subtracted. Due to the density and proximity of puncta, these were divided into three size groups of pixels; the group with the biggest size was not considered since it could only represent the same fibrillar processes. Then, images were normalized, and the threshold set, and puncta were counted automatically using ImageJ software. Means were determined for each experimental group, and the data were subjected to an unpaired Student’s *t*-test.

### Repetitive imaging of dentate gyrus mossy fiber boutons

Mouse entorhinal-hippocampal slice cultures were prepared and imaged as described before [86, 87]. At day 3 in vitro rAAV-**P**_hSYN_-EGFP, rAAV-**P**_hSYN_-rtTA and rAAV-**P**_tet_-bi-dsTeTxLC/TEV were locally injected into DG and rAAV-**P**_hSYN_-tdTOM into CA3 using glas pipettes. Two weeks later, i.e., 18-22 days in vitro, slice cultures on the filter inserts (Millipore, Germany) were transferred to a petri dish containing preheated (35 °C) imaging medium (NaCl 129 mM, KCl 4 mM, MgCl_2_ 1 mM, CaCl_2_ 2 mM, glucose 4.2 mM, HEPES 10 mM, Trolox 0.1 mM, streptomycin 0.1 mg/ml, penicillin 100 U/ml; pH 7.4; with sucrose, osmolarity was adjusted that matched the osmolarity of the culture medium). Filter inserts were secured by a custom-made titanium ring. The cultures were viewed with an upright Zeiss LSM Pascal confocal microscope. A 10x water immersion objective (0.3 NA, Zeiss, Germany) was used to visualize the culture at a low magnification to identify individual CA3 pyramidal neurons and mossy fibers. Then a 63x water immersion objective (0.95 NA; Zeiss, Germany) was used to image mossy fiber synaptic boutons. CA3 pyramidal neurons served as an orientation for re-identification across multiple imaging sessions. Up to 25 images were recorded per stack with an ideal Nyquist rate using the same imaging procedure and settings at the microscope for all consecutive time points. For analysis, 25 mossy fiber boutons were identified in in the middle of each confocal image stack and followed over time in maximum intensity 2D-projections of the following imaging time points.

### Data collection and analysis

The fEPSPs, the unrectified EMG activity of the recorded muscles, and 1-V rectangular pulses corresponding to CS, US, and electrical stimuli presented during the different experimental situations were acquired online through an 8-channel analog-to-digital converter (CED 1401-plus, CED, Cambridge, UK), and transferred to a computer for quantitative off-line analysis. Data were sampled at 8000 Hz (for fEPSP recordings) or 4000 Hz (for EMG recordings), with an amplitude resolution of 12 bits. Computer programs (Spike2 and SIGAVG from CED) were used to analyze field potentials and EMG activities. These programs allowed the quantification of the onset latency and area (mV’s) of the rectified EMG activity of the orbicularis oculi muscle with the aid of cursors. Field synaptic potentials (in mV) collected from the same session (*n* = 66) and animal were averaged, and the mean value of the slope (in mV/s) was determined for the rise time (i.e., the period of the slope between the initial 10% and the final 10% of the evoked field potential). Statistical analyses were performed using the Sigma Plot 11.0 package (Sigma Plot, San Jose, CA, USA) for a statistical significance level of *P* = 0.05. Unless otherwise indicated, mean values were calculated from 15 electrodes collected from three animals. Mean values are followed by their standard error (SEM). Collected data were analyzed using the one-way or two-way ANOVA test, with time or session as a repeated measure and contrast and non-parametric analysis when appropriate. Repeated-measures ANOVA allowed for checking the statistical differences of the same group across sessions. The student t-test was used when necessary.

## Supplementary information

Supplementary Table 1

## Data Availability

Upon request, all original data can be obtained from JMD-G (in vivo electrophysiology, behavior, and immunohistochemistry for vGLut and PSD95), AV (chronic imaging of mossy fiber puncta in cultured hippocampus slices), and MTH (characterization of tetanus toxin light chain technology in brain slices and biochemistry).
