# Supplementary material for: Dentate gyrus is needed for memory retrieval
Source: Mol Psychiatry. 2024 Apr 12;29(10):2939–50. doi: 10.1038/s41380-024-02546-0 (PMC11449802; doi:10.1038/s41380-024-02546-0)
Supplement: Supplementary file 1 — Supplementary Table 1 [file 41380_2024_2546_MOESM1_ESM.pdf]

Supplementary Table 1. Virus-injection coordinates.
Antero-posterior (AP), lateral (L) and depth (D). For control and unilateral Tet infected animals the lateral coordinate used was only the negative values. For bilateral infected animals the lateral coordinates used were both, negative and positive values to cover both hippocampi. The volume of viral cocktail used at every hippocampal site was 0.75 µl, and the injection process was performed at a low speed to prevent tissue damage.

Injection point	AP coordinate (mm)	L coordinate (mm)	D coordinate (mm)
1	3	±2	4.7
2	3	±3	4.5
3	4	±3	4.5
4	5	±4	4
5	5	±5	4.5
6	6	±5	4.3
7	6	±6.5	4.7
8	7	±6	5.3
9	7	±8.3	6.2
